# HIV and syphilis prevalence trends among men who have sex with men in Guangxi, China: yearly cross-sectional surveys, 2008–2012

**DOI:** 10.1186/1471-2334-14-367

**Published:** 2014-07-03

**Authors:** Xiaofang Wang, Guanghua Lan, Zhiyong Shen, Sten H Vermund, Qiuying Zhu, Yi Chen, Kaveh Khoshnood, Zunyou Wu, Zhenzhu Tang

**Affiliations:** 1 Institute of HIV/AIDS Prevention and Control, Guangxi Center for Disease Control and Prevention, No. 18 Jinzhou Rd, Qingxiu District, Nanning 530028, Guangxi, PR China; 2 National Center for AIDS/STD Control and Prevention, Chinese Center for Disease Control and Prevention, Beijing 102206, China; 3 Vanderbilt Institute for Global Health and Department of Pediatrics, Vanderbilt University School of Medicine, Nashville, TN 37240, USA; 4 Yale School of Public Health, Yale University, New Haven, CT 06520, USA

**Keywords:** HIV, Syphilis, Prevalence trends, MSM, China, Guangxi Province

## Abstract

**Background:**

Men who have sex with men (MSM) represent the fastest growing key population for incident HIV cases in China. We examined five consecutive years of HIV and syphilis prevalence and risk factors data among MSM in Guangxi Province with the second highest estimated number of people living with HIV/AIDS (PLWHAs) in China in 2011.

**Methods:**

We collected demographic and behavioral data from national sentinel surveillance and HIV/syphilis blood samples in five annual cross-sectional surveys from 2008 to 2012. We analyzed HIV and syphilis prevalence trends stratified by social/behavioral characteristics.

**Results:**

HIV prevalence climbed steadily from 1.7% (95% confidence interval [CI]: 1.0 to 3.0) in 2008 to 3.7% (95% CI: 3.0 to 5.0) in 2012. Syphilis prevalence increased steadily from 5.1% (95% CI: 4.0 to 6.0) in 2008 to 8.4% (95% CI: 7.0 to 10.0) in 2012. HIV prevalence rose notably among MSM who were ≤25 years of age, never married, did not engage in sexual intercourse with women in the past six months, and had not been tested for HIV in the past year. Syphilis prevalence rose notably among MSM who were >25 years of age, ever married or living with a partner, and engaged in sexual intercourse with women in the past six months. HIV prevalence was much higher in MSM with current syphilis than without. Finally, current syphilis was the most significant predictor of HIV infection, and age was the most significant predictor of syphilis infection.

**Conclusions:**

HIV and the syphilis prevalence expansion among MSM suggest an urgent public health prevention challenge for Guangxi provincial health officials. Risk factors for each infection differed such that all MSM, each of whom might be at risk of HIV, syphilis or both, should be targets for heavy intervention.

## Background

The global HIV epidemic continues to expand disproportionately among men who have sex with men (MSM) [1]; the prevalence ranging from 3-25% [1,2]. High HIV prevalence has been demonstrated among MSM in other regions of Asia [3], such as India (14.7%) and Thailand (24.6%). Sentinel surveillance in China in 2012 showed that the national HIV prevalence among MSM was 6.7% [4], fully 134 times higher than 0.05% in China’s general population [5]. Data from the 2011 surveillance system report indicate a much higher prevalence among MSM in some cities: 16.3% in Chengdu (Sichuan Province in the southwest), 12.5% in Zhengzhou (Henan Province in the center), and 10.3% in Harbin (Heilongjiang Province in the northwest) [6].

Although heterosexual transmission is mainly responsible for the spread of HIV in recent years in China, the prevalence of HIV among MSM is rising at an alarming rate [7] and the epidemic is in a rapid expansion phase [1]. Sentinel surveillance data in five provincial capitals in China showed that the percent of HIV-positive MSM had increased annually from 0.9% in 2003 to 6.3% in 2011 [6]. Of the newly reported HIV/AIDS cases nationwide, the proportion of MSM cases climbed from 15% in 2011 to 21.1% in 2012.

Syphilis likely increases the sexual transmission efficiency of HIV [1,8-10]. In China, national surveillance data from 2005 through 2011 showed syphilis prevalence among MSM was around 10% [6] in some provincial capitals. The largest study [7] of MSM (n = 47,321) and HIV and syphilis prevalence in China showed a correlation between HIV and syphilis infection, similar to findings from Chinese cohort studies [11,12]. There are a limited number of studies reporting HIV and syphilis prevalence trends among MSM in China, restricted to five other provincial capitals [6] or cities experiencing the highest HIV prevalence among the population [6,13,14].

Guangxi Province is located along a drug trafficking route, which originates from the Golden Triangle, passes through the northern provinces of Vietnam, and finally into Hong Kong and the rest of the world [15]. As the province with the second highest estimated number of people living with HIV/AIDS among 31 provinces in China in 2011 [5], Guangxi has been the focus of study for its heterosexual and needle sharing transmission routes [16]. In contrast, the HIV epidemic among MSM in Guangxi has not been well-studied though surveillance data suggest a growing contribution of MSM to the HIV epidemic. In this study, we analyzed five consecutive years’ of HIV and syphilis prevalence trends and risk factors among Guangxi MSM. The results of this study will assist in guiding HIV-related prevention and intervention efforts to effectively control the HIV epidemic in this province and beyond.

## Methods

### Study design

We reviewed and analyzed data from five cross-sectional surveys as part of the national sentinel surveillance [17] conducted yearly from 2008 through 2012 in the four largest cities in Guangxi Province (southern China): Nanning, Liuzhou, Guilin, and Beihai. Persons eligible for the study were men at least 18 years of age who reported at least one sexual encounter with a male sex partner in the previous year. Eligibility also included the ability to complete the survey and provide informed consent. Subjects were recruited by local Centers for Disease Control and Prevention (CDC) staff in partnership with local community-based organizations (CBO) via internet-based recruiting (internet dissemination through local gay websites and QQ chat room groups--QQ is the most widely used chat room software in China, the most well-known local gay man chat room, and well-known local gay men activist micro blogs.), and peer referral. Study information, including contact information, survey location and duration of the study, was publicized as an advertisement via internet. Through the advertisement, MSM were invited for a face-to-face interview and free HIV and syphilis tests. In 2011, due to the suspension of funding from The Global Fund to Fight AIDS, Tuberculosis and Malaria [18], recruitment strategy changed with more participants recruited from gay friendly community sites compared to internet-based sites.

The survey questionnaire included basic demographic characteristics including age, education, employment, marital status, resident status of Guangxi province (VS. migrant), use of illegal drugs (including heroin, cocaine, methamphetamine, ketamine, marijuana, and opium), prior HIV testing, and condom use with men and, when applicable, women. Trained interviewers, mainly local CDC staff members, were given a detailed protocol for the survey, conducted face-to-face interviews in private rooms at the CDC or the local MSM community based organizations (CBOs).

Blood samples were collected from all subjects for serologic testing for HIV and syphilis, regardless of self-reported HIV infection status. Subjects who tested positive were contacted by CDC staff for confirmatory testing. Counseling was provided before and after testing. HIV screening was conducted using enzyme-linked immunoassay (ELISA) with Western blot test confirmation, as per the China CDC national protocol. For syphilis, all specimens were tested with ELISA and rapid plasma reagin (RPR) testing in parallel, and specimens with reactivity on both tests were considered as having “current syphilis”. Participants who tested HIV or syphilis positive were referred to the National AIDS Program via an appropriate local hospital or clinic, while men who were uninfected were informed where they could access appropriate health services when needed.

### Statistical analyses

We used the Chi square test for assessing categorical associations of demographic and behavioral characteristics with HIV and syphilis over the five years. The Chi square test for linear trend examined HIV and syphilis prevalence trends over time. Both tests were included to account for some variables may have rising or falling trends over the years while they were not significantly different from each other. Additionally, to verify that results from the trend analysis were not affected by differences in the 2011 sampling approach, we repeated the trend analysis on every other sample, and compared the P-values with those of the five year sample.

We further examined HIV and syphilis prevalence trends for different demographic strata. Multivariate predictors were assessed with logistic regression analyses using HIV or syphilis as the dependent variables. We first did a univariate analysis, and then included the variables with *p* value <0.1 in the multivariate analysis. All *p* values are 2-sided. Data analysis was performed using SPSS software (version 17.0; SPSS Inc, Chicago, IL, USA).

### Ethical approval

We obtained ethical approval from the Institutional Review Board of Guangxi CDC for the study. Subjects provided signed informed consent and were assigned unique identification numbers, to prevent testing the same man twice in the same year. Participants were provided RMB 50 (50 yuan ≈ US$8) as compensation for transportation costs. Patients testing positive for any infection were referred to treatment, while men who were uninfected were informed where they could access future appropriate health services when needed.

## Results

The numbers of enrolled MSM in the five consecutive years (2008–2012) were 1146, 1351, 1257, 1314, and 1301 respectively. For each survey year, MSM were recruited in the same three to four months, from April to July. HIV prevalence was 1.7% (95% confidence interval [CI]: 1.0 to 3.0) in 2008, 1.3% (95% CI: 1.0 to 2.0) in 2009, 3.2% (95% CI: 2.0 to 4.0) in 2010, 2.7% (95% CI: 2.0 to 4.0) in 2011, and 3.7% (95% CI: 3.0 to 5.0) in 2012. The rise in HIV over time was coincident with an increase in syphilis prevalence: 5.1% (95% CI: 4.0 to 6.0) in 2008, 6.7% (95% CI: 5.0 to 8.0) in 2009, 7.9% (95% CI: 6.0 to 9.0) in 2010, 6.2% (95% CI: 5.0 to 7.0) in 2011, and 8.4% (95% CI: 7.0 to 10.0) in 2012 (Table 1).

**Table 1:** Changes in demographics and behaviors among men who have sex with men in Guangxi, 2008 to 2012

	**2008 (N = 1146)**	**2009 (N = 1351)**	**2010 (N = 1257)**	2011* (N = 1314)	**2012 (N = 1301)**		
**Variable**	**% (n)**	**% (n)**	**% (n)**	**% (n)**	**% (n)**	***p***	***p *** **trend**
Age							
≤25	42.9(492)	38.3(517)	36.7(461)	28.6(376)	44.2(575)	<0.001	0.15
>25	57.1(654)	61.7(834)	63.3(796)	71.4(938)	55.8(726)		
Current marital status							
Never married	70.9(813)	70.6(954)	70.0(879)	52.2(686)	77.9(1013)	<0.001	0.82
Married	21.8(250)	24.3(328)	25.2(316)	38.6(507)	18.4(240)		
Live with a partner	3.5(40)	1.3(17)	1.8(23)	3.7(48)	0.8(10)		
Divorced/widowed	3.8(43)	3.8(52)	3.0(38)	5.5(72)	2.9(38)		
Registered Guangxi resident							
Yes	83.5(957)	87.4(1181)	87.2(1095)	90.2(1183)	87.8(1142)	<0.001	**<0.001**
No, i.e., a migrant	16.5(189)	12.6(170)	12.8(161)	9.8(129)	12.2(159)		
Education							
Junior high school or lower	24.3(278)	29.5(398)	27.0(339)	16.1(211)	20.0(260)	<0.001	**<0.001**
Senior high school	36.8(422)	36.9(499)	38.6(485)	44.6(586)	37.6(489)		
College or higher	38.9(446)	33.6(454)	34.4(433)	39.3(517)	42.4(552)		
Had sex with male in the past 6 months							
Yes	77.9(876)	82.6(1109)	72.7(906)	83.3(1094)	67.2(873)	<0.001	**<0.001**
No	22.1(248)	17.4(234)	27.3(341)	16.7(220)	32.8(426)		
Used condom in the last male-male sex							
Yes	58.1(504)	66.1(731)	74.4(660)	80.3(874)	76.8(667)	<0.001	**<0.001**
No	41.9(364)	33.9(375)	25.6(227)	19.7(214)	23.2(201)		
Used condom with male in the past 6 months							
Yes	84.4(734)	91.3(1011)	90.9(808)	95.7(1045)	94.6(820)	<0.001	**<0.001**
No	15.6(136)	8.7(96)	9.1(81)	4.3(47)	5.4(47)		
Had sex with female in the past 6 months							
Yes	22.0(250)	22.4(302)	21.1(263)	28.3(372)	14.6(190)	<0.001	**0.01**
No	78.0(885)	77.6(1046)	78.9(983)	71.7(942)	85.4(1110)		
Used condom in the last sex with female**							
Yes	39.3(96)	46.0(138)	39.6(95)	80.1(298)	46.3(88)	<0.001	**<0.001**
No	60.7(148)	54.0(162)	60.4(145)	19.9(74)	53.7(102)		
Used condom with female in the past 6 months**							
Yes	56.4(137)	66.3(199)	68.1(171)	89.8(333)	64.0(121)	<0.001	**<0.001**
No	43.6(106)	33.7(101)	31.9(80)	10.2(38)	36.0(68)		
Ever used illegal drugs							
Yes	1.7(19)	1.0(13)	1.0(12)	0.1(1)	0.2(3)	<0.001	**<0.001**
No	98.3(1127)	99.0(1335)	99.0(1245)	99.9(1312)	99.8(1298)		
Received peer education in the past year							
Yes	45.1(516)	60.9(823)	66.6(836)	61.4(804)	53.3(694)	<0.001	**0.001**
No	54.9(629)	39.1(528)	33.4(419)	38.6(506)	46.7(607)		
Tested for HIV in the past year							
Yes	30.8(353)	53.0(716)	62.7(788)	63.0(826)	54.3(707)	<0.001	**<0.001**
No	69.2(793)	47.0(635)	37.3(468)	37.0(485)	45.7(594)		
HIV antibody positive***							
Yes	1.7(20)	1.3(17)	3.2(40)	2.7(35)	3.7(47)	<0.001	**<0.001**
No	98.3(1126)	98.7(1334)	96.8(1205)	97.3(1264)	96.3(1233)		
Current syphilis infection****							
Yes	5.1(59)	6.7(90)	7.9(99)	6.2(81)	8.4(109)	0.01	**0.01**
No	94.9(1087)	93.3(1261)	92.1(1158)	93.8(1233)	91.6(1192)		

*The sample in 2011 is slightly different.

**Only among men reporting sex with a female in the past 6 months.

***95% confidence intervals for HIV point prevalence: 2008 (1.0, 3.0), 2009 (1.0, 2.0), 2010 (2.0, 4.0), 2011 (2.0, 4.0), 2012 (3.0, 5.0).

****95% confidence intervals for syphilis point prevalence: 2008 (4.0, 6.0), 2009 (5.0, 8.0), 2010 (6.0, 9.0), 2011 (5.0, 7.0), 2012 (7.0, 10.0).

Note: The p values in bold were statistically significant trends which were highlighted to make them convenient to be identified.

There were a number of statistically significant changes in demographic characteristics over the five-year period (Table 1). The year 2011 (the year with proportionately fewer internet-recruited men) had apparent anomalies for both HIV and syphilis prevalence, as well as some socio-demographic indicators. Linear trends over time were typical, with the exception of age and current marital status. A trend analysis of every other sample showed that most variables had similar P-values under both testing methods (results not shown).

MSM >25 years of age accounted for the largest subgroup in 2011 (71.4%) alone, but was near 60% in the other years. Also different in 2011 alone was the proportion of men who were currently married: 38.6% in 2011 compared to about 20% in the other years. Differences were also noted in the proportion of men who were officially registered Guangxi residents, varying from 83.5% in 2008, to 90.2% in 2011, and 87.8% in 2012. MSM having received education of senior high school or higher were fewer in earlier than in later years (Table 1).

Condoms were reported to be used more frequently in later years. Having sex with a male without a condom at the time of last sexual encounter in the past six months decreased from 41.9% in 2008 to 19.7% in 2011 and 23.2% in 2012. This salutary trend was also noted for MSM who never used a condom with a male in the past six months, decreasing from 15.6% in 2008 to 5.4% in 2012. MSM who had a sexual encounter with a female in the past six months accounted for 20-30% of the sample in all years except 2012 (14.6%). In the subset of MSM who also had sex with women, having sex without a condom at the time of last sexual encounter with a female in the past six months decreased slightly from 60.7% in 2008 to 53.7% in 2012, though the 2011 data was highly disparate (19.9%). Men who never used a condom with a female in the past six months decreased from 43.6% in 2008 to 36.0% in 2012, with an atypical nadir of 10.2% in 2011. In addition, condom use with men in the last sexual encounter in the subset of MSM >25 years of age increased from 52.3% in 2008 to 83.1% in 2011and 76.7 in 2012, while in younger subset increased modestly from 65.5% in 2008 to 71.1% in 2012 (not shown in tables).

History of ever abusing drugs remained at a very low level and decreased from 1.7% in 2008 to 0.2% in 2012. The percentage of participants receiving peer education in the past year increased consistently from 45.1% in 2008 to 66.6% in 2010, falling to 53.3% in 2012. MSM reporting having been tested for HIV in the past year increased from 30.8% in 2008 to 60.3% in 2011 and 54.3% in 2012.

In the subset of men who had sexual intercourse with a woman in the past six months, condom use with men was significantly associated with condom use with women. Fully 81.9% who used a condom in their last sexual encounter with a female also used a condom in their last sexual encounter with a male, while 88.6% who used condoms with females in the past six months also used condoms with males.

HIV prevalence rose over time in both younger and older age groups (Table 2), with an especially high prevalence noted in younger men in 2011. A rising trend for HIV prevalence was most notable among MSM who had never married, were officially registered Guangxi residents, and/or did not report a sexual encounter with a female in the past six months. A rising HIV prevalence trend was seen for MSM in all subcategories of education, and whether or not they had had sex with men in the past six months, had received peer education in the past year, had been tested for HIV in the past year, or were currently infected with syphilis.

**Table 2:** Changes in HIV prevalence stratified by demographic/risk characteristics among men who have sex with men in Guangxi, 2008 to 2012

	**2008**	**2009**	**2010**	2011*	**2012**		
**Variable**	**% (n/N)**	**% (n/N)**	**% (n/N)**	**% (n/N)**	**% (n/N)**	***P***	***p *trend**
Age							
≤25	1.6(8/492)	1.4(7/517)	2.2(10/461)	4.3(16/376)	2.7(15/575)	0.04	**0.03**
>25	1.8(12/654)	1.2(10/834)	3.8(30/796)	2.0(19/938)	4.5(32/726)	<0.001	**0.001**
Current marital status							
Never married	1.5(12/813)	1.5(14/954)	2.5(22/879)	3.6(24/686)	3.9(39/1013)	0.001	**<0.001**
Other	2.4(8/333)	0.8(3/397)	4.8(18/377)	1.8(11/627)	2.8(8/288)	0.004	0.58
Registered Guangxi resident							
Yes	1.5(14/957)	1.2(14/1181)	2.9(32/1095)	2.8(33/1183)	3.7(41/1142)	<0.001	**<0.001**
No, i.e., a migrant	3.2(6/189)	1.8(3/170)	5.1(8/161)	1.6(2/129)	3.8(6/159)	0.37	0.75
Education							
Junior high school or lower	1.4(4/278)	1.3(5/398)	4.2(14/339)	2.4(5/211)	3.9(10/260)	0.05	**0.03**
Senior high school	1.4(6/422)	0.8(4/499)	4.0(19/485)	2.4(14/586)	3.1(15/489)	0.01	**0.02**
College or higher	2.2(10/446)	1.8(8/454)	1.6(7/433)	3.1(16/517)	4.1(22/552)	0.09	**0.02**
Had sex with male in the past 6 months							
Yes	1.9(17/876)	1.4(16/1109)	2.8(25/906)	2.8(30/1094)	3.7(32/873)	0.02	**0.002**
No	0.8(2/248)	0.4(1/234)	4.4(15/341)	2.3(5/220)	3.6(15/426)	0.01	**0.02**
Used condom in the last male-male sex**							
Yes	2.4(12/504)	1.5(11/731)	2.9(19/660)	2.5(22/874)	3.5(23/667)	0.19	0.08
No	1.4(5/364)	1.3(5/375)	2.7(6/227)	3.8(8/214)	4.5(9/201)	0.06	**0.004**
Used condom with male in the past 6 months**							
Yes	2.2(16/734)	1.6(16/1011)	3.0(24/808)	2.8(29/1045)	3.6(29/820)	0.07	0.02
No	0.7(1/136)	0(0/96)	1.2(1/81)	2.1(1/47)	6.4(3/47)	0.04	0.01
Had sex with female in the past 6 months							
Yes	2.0(5/250)	0.7(2/302)	1.1(3/263)	1.9(7/372)	2.7(5/190)	0.43	0.34
No	1.6(14/885)	1.4(15/1046)	3.7(36/983)	3.0(28/942)	3.8(42/1110)	0.001	**<0.001**
Received peer education in the past year							
Yes	1.6(8/516)	1.2(10/823)	3.3(27/836)	1.8(14/804)	3.8(26/694)	0.002	**0.006**
No	1.9(12/629)	1.3(7/528)	3.1(13/419)	4.0(20/506)	3.5(21/607)	0.04	**0.008**
Tested for HIV in the past year							
Yes	2.0(7/353)	1.3(9/716)	2.8(22/788)	2.0(16/826)	3.6(25/707)	0.04	**0.03**
No	1.6(13/793)	1.3(8/635)	3.9(18/468)	4.0(19/485)	3.7(22/594)	0.003	**0.001**
Current syphilis infection							
Yes	6.8(4/59)	3.3(3/90)	8.3(8/99)	14.1(11/81)	14.2(15/109)	0.06	**0.008**
No	1.5(16/1087)	1.1(14/1261)	2.8(32/1158)	2.0(24/1233)	2.7(32/1192)	0.009	**0.009**

*The sample in 2011 is slightly different.

**Only among men reporting sex with a male in the past 6 months.

Note: The p values in bold were statistically significant trends which were highlighted to make them convenient to be identified.

Current syphilis prevalence rose over time in the older age cohorts, those ever married or living with a partner, officially registered Guangxi residents, education of senior high school or lower, had no sexual encounters with men in the past six months, had a sexual encounter with a woman in the past six months, received no peer education in the past year, and had not received an HIV test in the past year (Table 3).

**Table 3:** Changes in syphilis prevalence with different demographic characteristics among men who have sex with men in Guangxi, 2008-2012

	**2008**	**2009**	**2010**	2011*	**2012**		
**Variable**	**% (n/N)**	**% (n/N)**	**% (n/N)**	**% (n/N)**	**% (n/N)**	***p***	***p *trend**
Age							
≤25	3.3(16/492)	5.2(27/517)	4.8(22/461)	5.1(19/376)	4.9(28/575)	0.60	0.33
>25	6.6(43/654)	7.6(63/834)	9.7(77/796)	6.6(62/938)	11.2(81/726)	0.002	**0.02**
Current marital status							
Never married	5.2(42/813)	6.8(65/954)	6.9(61/879)	5.7(39/686)	6.7(68/1013)	0.48	0.44
Other	5.1(17/333)	6.3(25/397)	10.1(38/377)	6.7(42/627)	14.2(41/288)	<0.001	**0.001**
Registered Guangxi resident							
Yes	4.9(47/957)	6.9(81/1181)	7.0(77/1095)	5.9(70/1183)	8.2(94/1142)	0.03	**0.02**
No	6.3(12/189)	5.3(9/170)	13.7(22/161)	8.5(11/129)	9.4(15/159)	0.06	0.15
Education							
Junior high school or lower	4.0(11/278)	7.3(29/398)	8.0(27/339)	8.5(18/211)	10.0(26/260)	0.10	0.01
Senior high school	4.7(20/422)	6.2(31/499)	8.9(43/485)	6.3(37/586)	9.2(45/489)	0.04	**0.02**
College or higher	6.3(28/446)	6.6(30/454)	6.7(29/433)	5.0(26/517)	6.9(38/552)	0.75	0.94
Had sex with male in the past 6 months							
Yes	5.6(49/876)	6.7(74/1109)	8.4(76/906)	6.3(69/1094)	8.1(71/873)	0.09	0.10
No	4.0(10/248)	6.0(14/234)	6.7(23/341)	5.5(12/220)	8.9(38/426)	0.14	0.02
Used condom in the last male-male sex							
Yes	6.5(33/504)	7.3(53/731)	9.1(60/660)	6.2(54/874)	8.4(56/667)	0.19	0.56
No	4.4(16/364)	5.6(21/375)	7.0(16/227)	7.0(15/214)	7.0(14/201)	0.57	0.12
Used condom with male in the past 6 months							
Yes	5.9(43/734)	7.2(73/1011)	9.2(74/808)	6.2(65/1045)	8.2(67/820)	0.06	0.29
No	4.4(6/136)	0(0/96)	2.5(2/81)	6.4(3/47)	8.5(4/47)	0.08	0.15
Had sex with female in the past 6 months							
Yes	2.4(6/250)	3.0(9/302)	6.8(18/263)	6.7(25/372)	11.6(22/190)	<0.001	**<0.001**
No	6.0(53/885)	7.6(80/1046)	8.2(81/983)	5.9(56/942)	7.8(87/1110)	0.15	0.46
Received peer education in the past year							
Yes	7.4(38/516)	7.3(60/823)	8.0(67/836)	6.1(49/804)	8.1(56/694)	0.60	0.99
No	3.3(21/629)	5.7(30/528)	7.6(32/419)	6.3(32/506)	8.7(53/607)	0.002	**<0.001**
Tested for HIV in the past year							
Yes	7.4(26/353)	7.5(54/716)	8.0(63/788)	6.7(55/826)	8.6(61/707)	0.68	0.67
No	4.2(33/793)	5.7(36/635)	7.7(36/468)	5.4(26/485)	8.1(48/594)	0.02	**0.006**

*The sample in 2011 is slightly different.

Note: The p values in bold were statistically significant trends which were highlighted to make them convenient to be identified.

Significant independent predictors for HIV infection included current syphilis infection, later survey years of 2010 and 2012, and no sexual encounters with a female in the past six months (Table 4). Significant independent predictors for syphilis infection included age >25 years, officially registered Guangxi residents, never used a condom with male in the past six months, and did not have a sexual encounter with a female in the past six months (Table 5).

**Table 4:** Logistic regression of HIV infection among men who have sex with men in Guangxi (surveys from all 5 years, 2008–2012)

		**Infection**	**Adjusted odds**	
**Variable**	**N**	**n(%)**	**Ratio OR (95% CI)**	**P-value**
HIV infection by year				0.001
2008	1146	20(1.7%)	1	
2009	1351	17(1.3%)	0.72(0.37,1.39)	0.32
2010	1245	40(3.2%)	1.78(1.02,3.12)	0.04
2011	1299	35(2.7%)	1.59(0.90,2.81)	0.11
2012	1280	47(3.7%)	2.06(1.20,3.55)	0.01
Had sex with female in the past six months				0.03
No	4924	135(2.7%)	1	
Yes	1372	22(1.6%)	0.60(0.38,0.95)	
Current syphilis infection				<0.001
No	5892	118(2.0)	1	
Yes	429	41(9.6%)	5.07(3.49,7.37)	

Note: Variables of HIV infection by year, had sex with female in the past six months, and current syphilis infection were included in the multivariate model.

**Table 5:** Logistic regression of syphilis infection among men who have sex with men in Guangxi (surveys from all 5 years, 2008–2012)

		**Infection**	**Adjusted Odds**	
**Variable**	**N**	**n(%)**	**Ratio OR (95% CI)**	**P-value**
Age				<0.001
≤25	2421	112(4.6%)	1	
>25	3948	326(8.3%)	1.83(1.42,2.36)	
Registered Guangxi resident				0.04
No	808	69(8.5%)	1	
Yes	5558	369(6.6%)	0.73(0.54,0.98)	
Used condom with male in the past 6 months				0.008
No	4431	323(7.3%)	1	
Yes	407	15(3.7%)	0.49(0.29,0.83)	
Had sex with female in the past six months				0.01
No	4966	357(7.2%)	1	
Yes	1378	80(5.8%)	0.69(0.52,0.92)	

Note: Variables of age, registered Guangxi resident, used condom in the last male-male sex, used condom with male in the past 6 months, had sex with female in the past six months, and syphilis infection by year were included in the multivariate model.

## Discussion

In this study, we found significantly rising trends of both HIV and syphilis prevalence among MSM in the four largest cities in Guangxi Province, China. Similar serial cross-sectional studies reported from China are few and often do not provide multi-year data [13,14,19]. Our finding of the rising prevalence exists in the context of increasing self-reported condom usage over time, similar to the finding of a recent meta analysis [20] of HIV risk reduction intervention studies among Chinese MSM. This is plausible because as background prevalence increases, MSM may have a higher risk of encountering an infectious partner, even if their own risk behaviors diminish somewhat; that is, the reported increased condom usage were not sufficient to offset the effects of elevated risk of infection [21].

The value of serial cross sectional surveys is apparent: HIV prevalence almost tripled over just five years, although absolute Guangxi rates for MSM were a bit lower than that estimated nationwide [5] from 5% in 2008 to 6.7% in 2012 or other selected cities [6,9,12] in China. The upward trend is similar to that seen in some other Chinese cities and nationwide in time periods proximate to those in our study [6,13,19]. The epidemic among MSM in Guangxi may be more serious than our findings indicate due to dual stigma of male-male sexual behavior and HIV infection. Additionally, HIV/AIDS prevention programs targeting MSM were only first carried out in Guangxi in 2006 which is later in comparison to other provinces or municipalities reporting a higher prevalence among MSM than Guangxi [20]. Over time, the MSM population in Guangxi may likely continue to disproportionally contribute to new HIV and syphilis cases if there remains a dearth in targeted prevention strategies. Guangxi has a significant opportunity to control the HIV epidemic among MSM before becoming more serious as in other areas in China.

In our study, MSM who reported no sex with women in the past six months were more likely to be HIV infected than those who had. This is similar to the finding reported in a study of southern African MSM engaging in heterosexual intercourse in the previous six months [22]. The men who report having sex with both men and women in Guangxi may play a role as a bridge of transmitting HIV to their female sex partners, but the risk is lower than among men who are exclusively having sex with men.

Our study found HIV prevalence to be much higher in MSM with current syphilis and syphilis was shown as a probable risk factor for HIV infection. That syphilis may facilitate HIV transmission among MSM is not surprising, and reinforces the need for aggressive public health measures to combat sexually transmitted infections [20,23,24].

The syphilis prevalence shown in our study was somewhat lower than that reported in previous Chinese studies: a meta-analysis [25] and the largest study [7] of HIV and syphilis infection among MSM in China reported syphilis prevalence of 9.1% and 11.8% respectively. However, syphilis prevalence increased year by year, notably among MSM who were older, married, officially registered local residents, less educated, and had a sexual encounter with a female in the past six months--all of which probably demonstrated an increasing risk of syphilis transmission from men having sex with both sexes to their female and male partners. Even though the probability of HIV transmission is much higher through unprotected receptive anal sex than unprotected vaginal sex [1], this finding suggests a possible increase in risk of HIV infection among the female sexual partners of the MSM. Therefore, to prevent further HIV transmission among MSM and their female sexual partners, timely treatment and prevention of syphilis warrants more attention. MSM >25 years are a key sub-population of concern for syphilis intervention [7].

Our finding that suboptimal MSM condom use was the rule with females was also demonstrated in a systematic review [26]. HIV interventions for MSM seldom promote condom use with their female sexual partners [27], accompanied by the traditional thinking that condoms with females were used mainly for contraception rather than an HIV/STD prevention strategy [28]. Loyalty and trust also facilitate sex without a condom [29]. Most MSM who used a condom with a female also used a condom with a male. HIV interventions among MSM should highlight the importance of condom use during any sexual encounters with either female or male partners.

The rising trend of condom use among the MSM population with male in the last sexual encounter in the five years of the study was more pronounced among MSM >25 years. This could indicate that MSM ≤25 years who are more sexually active are more likely to omit condom use or intentionally take risks, despite their knowledge about HIV transmission and prevention practices. This might indicate that the attitude toward HIV infection among the younger generation is changing: HIV is no longer viewed as a death sentence due to the knowledge of a long latent period and successful results with anti-retroviral treatment (ART). Also, to the young MSM, the desire for immediate gratification may be prioritized over health at the moment of sexual intercourse, especially if sex without any barriers is a replacement for lack of intimacy and emotion for them.

Demographic and behavioral characteristics in 2011 were significantly different from those of other years. Since there was a one year suspension of financing from the Global Fund [18] in 2011, less outreach was conducted by local CBOs among young MSM, particularly through the internet-based gay community where CDC staff have little access. That there was an asymmetry in recruiting in the 2011 year only suggests that trends may be distorted by alternative recruitment biases in that year.

HIV and syphilis prevalence increased significantly in the subcategory of MSM who had not tested for HIV in the previous year, while the trend was not substantial or did not exist in the subcategory of MSM who had tested for HIV in the previous year. HIV testing is a crucial strategy to effectively prevent HIV transmission among MSM [30] as early identification of infection enables timely treatment and increases knowledge of HIV status in order to prevent infecting others [31]. Large scale HIV testing also helps and contributes to increasing awareness of HIV prevention by promoting safe sexual behaviors including condom use, and it has shown to reduce HIV incidence rates among MSM [32-34]. Frequent HIV testing among MSM results in increased condom use and other protective sexual behaviors [35]. The testing rates in our study surpassed the national level in 2011 of 50.4% [36], but remain too low. This calls for more interventions to expand HIV testing and promote regular testing among the MSM population in the province.

### Strengths and limitations

Our study has strengths in that sample sizes were substantial and serial cross-sectional surveys were conducted with similar methods, with some deviation in 2011. Limitations noted include a potential overlap between yearly samples. Due to the anonymity of the survey respondents, we are unable to adjust for possible repeated measures from any survey respondent from any two or more years. Further, we cannot infer causality of HIV/syphilis infection and their predictors in a study sample that represents an aggregation of data from five yearly cross-sectional studies. Probable social desirability bias always exists in self-reported data which may have resulted in an underestimation of reported risk behaviors. In an attempt to minimize this bias, interviewers received training and the surveys were always conducted in private rooms or settings. Finally, because the sample only included large cities in the province, our results may not be generalizable to small cities or rural areas. Also, the marked difference in many of the sexual and demographic characteristics in 2011 when the recruitment strategy was changed demonstrated that recruitment strategies dictate sample representativeness. The sample in our study may not be generalizable to the whole MSM population. However, our comparatively large sample size at the provincial level and diverse sampling techniques reduces the probability that the sample is unrepresentative.

## Conclusions

Both the HIV and syphilis epidemics have expanded among MSM in Guangxi Province. Our study results illustrate the urgency for provincial HIV prevention and intervention strategies among this at risk population, including evidence-based policy decisions for program expansions. Comprehensive HIV prevention and intervention, supported and funded by local government, should include MSM-friendly HIV testing, syphilis treatment and prevention, peer education, CBO engagement and other HIV intervention measures for expanding HIV testing and condom use, and must be continuously strengthened for the MSM population in order to curb HIV epidemic in the province.

## Competing interests

The authors declare that they have no competing interests.

## Authors’ contributions

XFW performed analysis of the data and wrote the manuscript. GHL, QYZ and YC implemented the study and collected the data. ZYS and ZZT supervised the implementation of the study. SHV, KK and ZYW reviewed and revised the manuscript. All authors read and approved the final manuscript.

## Pre-publication history

The pre-publication history for this paper can be accessed here:

http://www.biomedcentral.com/1471-2334/14/367/prepub
